# Resistance of *Mycobacterium tuberculosis* to antibiotics in Lao PDR: first multicentric study conducted in 3 hospitals

**DOI:** 10.1186/1471-2334-13-275

**Published:** 2013-06-19

**Authors:** Vibol Iem, Silaphet Somphavong, Yves Buisson, Nicolas Steenkeste, Franck Breysse, Monique Chomarat, Phannasinh Sylavanh, Phouratsamy Nanthavong, Alain Rajoharison, Jean-Luc Berland, Phimpha Paboriboune

**Affiliations:** 1 Ministry of health, Centre d’Infectiologie Christophe Mérieux du Laos, Samsenthai Road, Kaoyot Village, Sisathanak District, Vientiane Capital, Lao PDR; 2 Institut de la Francophonie pour la Médecine Tropicale, Ban Kaognoth, rue Samsènethai, Vientiane Capital, Lao PDR; 3 Laboratoire de Bactériologie, Groupement Hospitalier Sud, Lyon, France; 4 The National Tuberculosis Control Program Center, Vientiane, Lao PDR; 5 Service de Pneumologie, Hopital Mahosot, Vientiane Capital, Lao PDR; 6 Laboratoire des Pathogènes Émergents, Fondation Mérieux, 21 Avenue Garnier, Lyon, 69007, France

**Keywords:** Multidrug resistant TB, Non Tuberculosis Mycobacteria, Multicentric

## Abstract

**Background:**

It is estimated that Lao People’s Democratic Republic (Lao PDR) ranks fifth among the seven countries most affected by TB in the WHO Western Pacific Region. However, because of late implementation of mycobacterial culture, no study on resistance to anti-TB drugs had been performed yet. The objective of this study was to document drug resistance rate among patients hospitalized for pulmonary TB in threeprovinces of Lao PDR.

**Methods:**

A cross-sectional study was conducted in three sites, one central and two regional hospitals, from April to November 2010. For each TB suspected patient sputum smear microscopy and culture on Lowenstein-Jensen media were performed. GenoType® MTBDRplus assay was used to test the susceptibility to isoniazid (INH) and rifampicin (RMP), GenoType® MTBDRsl for second-line drugs and GenoType® Mycobacterium CMAS for non-tuberculous mycobacteria (NTM).

**Results:**

Out of 104 positive culture on Lowenstein-Jensen, 87 (83.6%) were *M. tuberculosis* and 17 (16.4%) were NTM. Of 73 new TB cases, 5 isolates (6.8%) were resistant to INH. Of 14 previously treated cases, 2 isolates (14.3%) were resistant to INH and one isolate was XDR.

**Conclusion:**

Despite an overall rate of resistance still moderate, the frequency of mutations conferring INH monoresistance and identification of the first strain of XDR require strengthening surveillance of drug resistant tuberculosis in Lao PDR.

## Background

The control of tuberculosis (TB) in developing countries is hampered by the progression of drug-resistant TB, especially multi-drug resistance (MDR). MDR-TB strains are resistant to at least the first-line drugs isoniazid (INH) and rifampicin (RMP), which significantly reduces the therapeutic arsenal. The weighted rates of resistance in the world population are estimated among new cases at 17% for any resistance and 2.9% for MDR, and among previously treated cases at 35% for any resistance and 15.3% for MDR [1]. The World Health Organization (WHO) estimates that each year 440,000 new cases of MDR-TB are emerging globally, and 150,000 patients with MDR-TB die. More worrying is the emergence of extensively drug-resistant TB (XDR-TB) caused by MDR strains that also resist to fluoroquinolones and second-line anti-TB injectable drugs (amikacin, kanamycin or capreomycin). WHO estimates that 25,000 cases of XDR-TB emerge each year worldwide. Unfortunately, only a small fraction of patients infected by MDR or XDR-TB can access a screening by culture, drug-susceptibility testing and appropriate management [2]. The use of new rapid molecular tests for first-line drug resistance can dramatically reduce the delays to detect MDR-TB and adapt the treatment. The good performances of molecular line probe assays have led WHO to approve their use for rapid screening of patient at risk of MDR-TB [3].

In Lao PDR, the burden of TB remains high: it is the seventh leading cause of death in adults. In 2009, the prevalence of all forms of TB was estimated at 289/100 000, the incidence at 151/100 000 and the mortality at 24/100 000 [4]. The DOTS was implemented in 1995 by the National Tuberculosis Control Program (NTCP) [5]. The recommended treatment uses combinations of 5 drugs at fixed doses (S: streptomycin, R: rifampicin, H: isoniazid, Z: pyrazinamide, E: Ethambutol) corresponding to the following protocols: 2RHZE/6HE for new TB cases, 2RHZE/4RH for TB-HIV co-infections and 2RHZES/6RHZE for re-treatment. The therapeutic success rate of new smear positive cases exceeds 90% since 2005 and reached 93% in 2009. However, a retrospective cohort study of TB patients showed that the 85% survival rate was lower than the expected 91% from official statistics and that follow-up should be strengthened to improve patient compliance and allow contact tracing and detection of new cases [4,6]. The implementation of the first mycobacterial cultures in 2009 has made feasible the anti-TB drug susceptibility testing [7]. The creation of a national reference laboratory for TB allowed the realization of a first national prevalence survey in 2010–2011. But more efforts should be implemented to achieve the Millennium Development Goal 6 of halving TB prevalence and reverse its incidence by 2015 [8].

In this context, this study aimed to provide a preliminary assessment of the rate of *M. tuberculosis* resistance to isoniazid (INH), rifampicin (RMP) and second-line drugs in Lao PDR using a molecular line probe assay.

## Methods

### Design of the study

We conducted a cross-sectional study from April to November 2010 in a central hospital in Vientiane (Mahosot hospital) and two provincial hospitals (Thakhek and Luang Prabang). Patients enrolled in this study were directly admitted to one of the 3 hospitals and not transferred from another health care facility.

### Study population

All patients over 15 years attending the pneumology units with symptoms suggestive of TB were included, regardless of their HIV status, as well as patients given a TB treatment. These patients were considered (i) as new cases if never previously treated or beginning the treatment within the last month prior inclusion, (ii) as previously treated cases if the treatment started more than one month before the inclusion [2]. Inclusion criteria were cough for over two weeks, or bloody sputum, or suggestive radiological signs, irrespective to HIV infection. Chest radiography or tuberculin skin tests were not systematically performed.

### Specimen processing

Two sputum samples were collected for each patient, the first during the consultation, the second the next morning upon awakening. Screening for acid-fast bacilli (AFB) was locally performed in each hospital on Ziehl-Neelsen stained smears. Sputum were then stored at 4°C and sent to Rodolphe Mérieux Laboratory in Vientiane once a week for culture and drug susceptibility testing.

Specimens were digested and decontaminated by the N-acetyl-L-cystein-sodium hydroxide (NALC-NaOH) procedure [9], then inoculated on two slants of Lowenstein-Jensen medium incubated at 37°C. Cultures were examined once a week for 80 days. Any suspect colony confirmed as AFB by microscopy was identified and tested for antibiotic resistance.

### *Identification of* M. tuberculosis *and drug susceptibility testing*

When several cultures were positive for the same patient, only one was tested to detect drug resistances. The GenoType® MTBDR*plus* (Hain lifescience Gmbh, Germany) was used for *M. tuberculosis* complex identification and INH and/or RMP resistance detection according to manufacturer's instructions.

In case of resistance to INH and/or RMP, the Genotype® MTBDR*sl* (Hain lifescience Gmbh, Germany) was then performed to detect the resistances to second-line antibiotics.

Isolates not belonging to the *M. tuberculosis* complex were identified using the Genotype® Mycobacterium CM/AS (Hain lifescience Gmbh, Germany) that can differentiate 13 species of clinically relevant non-tuberculous mycobacteria (NTM).

### Statistical analysis

Data were entered with EPI data 3.1 and analyzed with Stata 8.0. The chi2 test was used to compare qualitative variables with a significance level of 0.05.

### Ethical clearance

This study was approved by the National Ethics Committee of Lao PDR and the National Tuberculosis Control Program Center (# 201/NECHR).

A written informed consent for participation in the study was obtained from participants. Only patients over 15 years old with signed informed consent were included in the study.

## Results

### Mycobacterial culture and identification

Eighty-seven isolates (83.6%) were identified as belonging to the *M. tuberculosis* complex, evenly distributed between the three sites (p=0.1). The 17 remaining strains (16.4%) were NTM of which 10 were identified as *M. fortuitum* (8)*, M. scrofulaceum* (1) and *M. abscessus* (1). Out of the 87 *M. tuberculosis* strains, 74 were isolated from new cases while 13 were isolated from previously treated cases (Table 1).

**Table 1:** Results of culture and identification

**Number of patients with**	**Total**	**Vientiane**	**Thakhek**	**Luang Prabang**	**p**
≥ 1 positive culture (%)	104	39 (37.5)	38 (36.5)	27 (26)	
*M. tuberculosis* complex	87	34 (39.1)	31 (35.6)	22 (25.3)	0.7
NTM*	17	5 (29.4)	7 (41.2)	5 (29.4)	

*NTM non-tuberculous mycobacteria (8 *M. fortuitum*, 1 *M. scrofulaceum*, 1 *M. abscessus* and 7 unidentified).

### Characteristics of patients

Among the 87 patients with at least one positive culture for M. tuberculosis, 34 (39.1%) lived in Vientiane, 31 (35.6%) in Thakhek and 22 (25.3%) in Luang Prabang. Sex ratio (M/F) was 1.3 and mean age 48 years. Most of the patients were new cases (85%). The 13(15%) patients previously treated or treated for more than 1 month were unevenly distributed among the three sites: 63% Vientiane, 19% in Luang Prabang and 19% in Thakhek. HIV status was only known for 5 patients; none of whom were HIV positive (Table 2).

**Table 2:** Characteristics of patients

	**Total**	**Mahosot**	**Thakhek**	**Luang Prabang**	**p**
	**87**	**34 (39%)**	**31 (36%)**	**22 (25%)**	
**Gender**					0.8
Male	50	21 (42)	17 (34)	12 (24)	
Female	37	13 (35.2)	14 (37.8)	10 (27)	
**Average age** (IC95%)	48 (45-52)	52 (45-59)	46 (41-51)	46 (38-55)	
**Age groups**					0.06
• ≥15-25< years	7	3 (42.9)	1 (14.2)	3 (42.9)	
• ≥25-55≤ years	48	14 (29.2)	23 (47.9)	11 (22.9)	
• > 55 years	32	17 (53.2)	7 (21.9)	8 (25)	
**TB history**					<0.4
Never treated or <1 months	74	27 (36.5)	28 (37.8)	19 (25.7)	
Previously treated or ≥ 1 months	13	7 (53.8)	3 (23.1)	3 (23.1	
**HIV status**					0.1
Positive	0	-	-	-	
Negative	5	1 (20)	4 (80)	0 (0)	
Unknown	82	33 (40.3)	27 (32.9)	22 (26.8)	

### Drug resistance

#### *Resistance to first line drugs*

Among the 87 *M. tuberculosis* isolates, 8 (9.2%) were found resistant: 7 mono-resistant to INH and 1 to both INH and RMP; 3 (13.6%) in Luang Prabang, 3 (9.7%) in Thakhek and 2 (5.9%) in Vientiane, without significant difference between the three sites (p=0.5). Five of them were isolated from new cases and were resistant to INH: two mutated in the *katG* gene (S315T), two in the *inhA* gene (C15T) and one in both genes *katG* (S315T) and *inhA* (C15T) (Table 3). The three remaining isolates were from previously treated cases: they were resistant to INH by S315T mutation in *katG* or by deletion of this gene. For this last strain (165 LP), none of the 4 *katG* locus probes on the strip reacted; further sequencing showed neither *katG* gene nor *katG* promoter could be amplified using several inner and outer primers, reflecting the complete deletion of this gene. To further analyze this strain, *rpoB*, *pncA*, *inhA* and *ahpC* genes were sequenced. No mutation was observed in *rpoB*, *pncA* and *inhA* genes, suggesting RMP and pyrazinamide susceptibility. The *ahpC* gene sequencing revealed the ambiguous base calls in the −51, -52 and −57 promoter positions. Therefore, amplicons were cloned and 20 of them were sequenced. Interestingly, the results showed the coexistence of three different mutational profiles, none with more than one mutated site. This strain was isolated from an 82-old patient who had received a TB treatment six years ago and was considered cured. He relapsed in 2012 and died quickly, before the culture results are positive. The third strain (175 TK) was also resistant to RMP by mutation in the *rpoB* gene (H526Y) and was therefore MDR (Table 3).

**Table 3:** **Mutation patterns of eight isolates of *M. tuberculosis***

**Patient number (hospital)**	**Previously treated case**	**Mutations conferring resistance to**				
		**INH**	**RMP**	**FLQ**	**AG/CP**	**EMB**
425 (LP)	No	katG S315T	-	-	-	-
		*inhA* C15T				
472 (TK)	No	*inhA* C15T	-	-	-	-
977 (MV)	No	katG S315T	-	-	-	-
454 (MV)	Yes	katG S315T	-	-	-	-
446 (LP)	No	*inhA* C15T	-	-	rrs C1402T	-
480 (TK)	No	katG S315T	-	-	rrs C1402T	-
165 (LP)	Yes	*katG* deleted	-	-	rrs C1402T	-
175 (TK)	Yes	katG S315T	rpoB H526Y	gyrA A90V	rrs C1402T	embB M306V
Total		8	1	1	4	1

*LP* Luang Prabang, *MV* Mahosot Vientiane, *TK* Thakhek, *INH* isionazid, *RMP* rifampycin, *FLQ* fluoroquinolones, *AG/CP* aminoglycosides, cyclic peptides, *EMB* ethambutol.

(−) Presence of all wild type probes and none mutation probe observed.

### Drug resistance

#### *Resistance to second-line drugs*

Out of the five new cases displaying resistance mutations, two INH-resistant strains were also resistant to aminoglycosides and cyclic peptides showing a C1402T mutation on the *rrs* gene. Out of the three previously treated cases, two strains were resistant to at least one second-line drug (Table 3).

The MDR-TB isolate (175-TK) displayed three further mutations: M306V in *embB* gene, A90V in *gyrA* gene and C1402T in *rrs* gene. This strain was sent to Korean Institute of Tuberculosis (KIT, Seoul, Korea) to perform a conventional antibiogram with the second line drugs. It turned out that this strain was indeed resistant to RMP, INH, ethambutol, fluoroquinolones, streptomycin and aminoglycosides/cyclic peptides, and it was therefore an XDR strain. The patient (175 TK) was a 31 years old woman, diagnosed pulmonary TB in 2004 and treated with a first line regimen (2RHZE/6HE) for 8 months. She was considered clinically cured with smear microscopy negative at fifth month of treatment. She relapsed in 2007 and received a second-line treatment (2RHZES/6RHZE) for 8 months followed by INH monotherapy for 3 years. In 2010, the diagnosis of chronic pulmonary TB resistant to treatment was bacteriologically confirmed. She then received a combination therapy of HZE, kanamycin and ofloxacin for 6 months (Table 4).

**Table 4:** History of the three previously treated patients

	**Patient number (hospital)**		
	**454 (MV)**	**165 (LP)**	**175 (TK)**
Gender / age (years)	M / 54	M / 82	F / 31
date of 1^st^ diagnosis of TB	2003	2004	2004
1^st^ anti-TB regimen	2RHZE/6HE	2RHZE/6HE	2RHZE/6HE
Outcome of 1^st^ TB treatment	recovery	recovery	failure
Date of TB relapse	2010	2012	2007
Date of initiation of treatment	2012	a	2007
Current anti-TB regimen	2RHZES	a	2RHZES/6RHZE
Duration of treatment	2 months	a	8 months + H 3 years
Compliance with TB treatment	unknown	a	high
Clinical response to treatment	poor	a	poor (b)

a: the patient died before the culture results and did not receive the second-line treatment.

b: chronic pulmonary tuberculosis resistant to treatment.

## Discussion

The recent implementation of mycobacterial culture in Laos PDR had made possible the necessary monitoring of resistance to anti-TB drugs. This multicenter study aimed to provide preliminary data on the rate of resistance to first-line anti-TB, especially INH and RMP. Patient enrolled were outpatients, not referred from peripheral clinics, thus not being at higher risk of drug resistant. The results show an overall rate of resistance of approximately 10%, homogeneously distributed among the 3 sites. It reached 6.8% among new cases and 21.4% among previously treated cases. Despite a sampling not as robust as national survey, this study shows similar rates compared to neighboring countries: Cambodia, 10% and 16.6% in the 2001–2002 national survey [10], and Myanmar, 10% and 30.2% in the 2002 national survey [11]. However, it remains well below the rates reported in Thailand, 20.9% and 53.6% in 1996 [12], in Vietnam, 26.3% and 62.9% in 2001 [13] and in China, 27.9% and 60.3% in a meta-analysis covering the period 1993–2011 [14].

All the resistant isolates of this study showed mutations conferring resistance to INH. The only isolate resistant to RMP, also carrying mutations conferring resistance to second-line antibiotics, came out to be an XDR. Even though no strictly MDR strain was isolated during the study, these results show the existence of resistance mutations in TB isolates that may lead to compromise the effectiveness of treatment regimens recommended by the NTCP.

This study presents a number of limitations. The patient sampling was performed in three large hospitals which do not represent the whole country. A recruitment in remote district hospitals where patient follow-up and compliance to DOTS are less easy to perform [6] might have revealed higher levels of resistance. The HIV status of patients was unknown for 92% of patients, indicating that the recommendations for routine screening of HIV-TB co-infection had not been yet systematically applied. The exclusive use of a molecular test for rapid species identification of mycobacterial isolates and for detection of antibiotic resistance may also lead to underestimate INH resistance. This line probe assay is part of rapid methods recommended by WHO for detection of MDR strains as a substitute for conventional methods which require 4 to 6 weeks. It can even be performed directly from sputum samples found positive at microscopy. Rapid detection of resistance to RMP is a major issue because it can be considered a surrogate marker for MDR strains [15]. According to a meta-analysis of 14 comparative studies for RMP and 15 for INH, performances of the GenoType MTBDR assay are considered satisfactory, except for the detection of INH resistance whose sensitivity approaches 90% with the MTBDRplus assay [16]. Some strains phenotypically resistant to INH are found genetically susceptible, possibly because of mutations involving genes other than *inhA* and *katG*[17]. Similarly, the MTBDRsl assay showed an excellent specificity in detecting resistance to second-line drugs, but a variable sensitivity, especially for the detection of resistance to fluoroquinolones and ethambutol [18,19]. The inclusion of new alleles is recommended in order to reduce the proportion of false negative results [20].

The eight resistant strains isolated in our study all showed a mutation conferring resistance to INH: 5 in *katG*, 2 in *inhA* and the remaining 1 in both *katG* and *inhA*. Interestingly, the three *inhA* promoter mutants were observed in new cases,. A study conducted during a 9-years period in San Francisco showed that strains with a *katG* S315T or *inhA* promoter mutation were more likely to spread than otherwise mutated strains [21]. However, mutations in the *inhA* promoter are more common among INH-monoresistant isolates than among MDR isolates [22]. It is in all cases a C15T mutation which is associated with a low level of resistance [23]. This mutation confers co-resistance to INH and ethionamide [24].

Of the 6 strains mutated in *katG*, 5 had the S315T mutation, currently considered as a risk factor for further development of MDR-TB [25]. The last one had a complete deletion of this gene, a mechanism rarely found associated with clinical INH-resistance [26,27]. By sequencing, the distinction of three clones each carrying one peculiar mutation in the *ahpC* gene is consistent with the hypothesis that up regulation mutations in the *ahpC* promoter compensate for the loss of catalase-peroxidase activity encoded by the *katG* gene [28].

The only case of RMP-resistance was due to a mutation H526Y in the *rpoB* gene. With codons 531 and 516, this point mutation is one of the most frequently associated with resistance to RMP [29].

Four strains, two from new cases and two from previously treated cases, exhibited a C1402T mutation in the *rrs* gene, presumed to confer resistance to aminoglycosides and cyclic peptides: this mutation was found associated with high levels of resistance to capreomycin, but with low levels of resistance to kanamycin and amikacin [30]. Regarding the two new cases, this mutation strongly suggests a primary resistance whose origin is unclear, the only aminoglycoside antibiotic widely used in Lao PDR as first-line treatment until the 2000s being the streptomycin.

Despite the progress made by the NTCP to achieve the WHO objectives, implementation of the DOTS strategy in Lao PDR is limited by the remoteness of the target population to health centers, the difficult follow-up of patients under TB treatment and the lack of compliance. The recent development of mycobacterial culture and the use of line probe assays have allowed identifying the species of the *M. tuberculosis* complex and, for the first time, to detect resistances to anti-TB drugs. This preliminary investigation shows that resistance mutations, especially against INH, are present among the new cases, some of which may evolve towards MDR.

The isolation of an XDR strain from a patient enrolled in a provincial hospital is the result of chance. This strain acquired resistance as a result of successive treatments; XDR being the consequence of adding only 2 drugs never received to a previous treatment failure, instead of 5 as recommended by WHO. In Cambodia, of 101 MDR strains isolated between 2007 and 2009, only one was found XDR [31]. However, this must sound like a threat because the XDR strains have the ability to spread in the population by intra-community transmission [32] and justifies the strengthening and extension of the laboratory network for tuberculosis in the different provinces of Lao PDR. The use of line probe assays such as MTBDR tests should now be recommended to rapidly detect and treat without delay the MDR-TB cases.

## Conclusion

The frequency of primary resistance to INH and identification of the first XDR strain strongly justify to strengthen and expand the screening of resistant TB in the provinces of Lao PDR.

## Competing interests

The authors declare that they have no competing interests.

## Authors’ contributions

VI, SS, JLB, NS, FB, MC, PS, PN, AR, YB, PP contributed to experimental procedures.

VI, SS, JLB, NS, MC, FB, PS, YB, PP drafted and critically revised the manuscript. All authors read and approved manuscript.

## Pre-publication history

The pre-publication history for this paper can be accessed here:

http://www.biomedcentral.com/1471-2334/13/275/prepub
